# Chromosome-Level Genome Assembly and Annotation of the Freshwater Snail *Sinotaia angularis* (O. F. Müller, 1774)

**DOI:** 10.3390/ani16131975

**Published:** 2026-06-26

**Authors:** Enjie Chua, Zhiqiang Wang, Jie Huang, Yanhong Wen, Xiaoyun Zhou, Fuguang Luo

**Affiliations:** 1 Guangxi Key Laboratory of Marine Environmental Disaster Processes and Ecological Protection Technology, College of Marine Sciences, Beibu Gulf University, Qinzhou 535011, China; chua.jie.6@gmail.com; 2 Liuzhou Aquaculture Technology Extending Station, Liuzhou 545006, China; wangzhiqiang2019@foxmail.com (Z.W.); huangjie_2026@foxmail.com (J.H.); wenyanhong888@163.com (Y.W.); 3 College of Fisheries, Huazhong Agricultural University, Wuhan 430070, China

**Keywords:** *Sinotaia angularis*, PacBio HiFi sequencing, Hi-C scaffolding, Viviparidae, freshwater snail

## Abstract

*Sinotaia angularis* is a freshwater viviparid snail with limited genomic resources. Here, we report a chromosome-level reference genome generated using PacBio HiFi sequencing and Hi-C scaffolding, with mitochondrial marker -based screening (16S rRNA and COI (cox1)) and phylogenetic analysis supporting the taxonomic assignment. A total of 74.08 Gb of HiFi reads were obtained, providing approximately 65.7-fold genome coverage. The final assembly spans 1.127 Gb, with a scaffold N50 of 141.87 Mb and a GC content of 34.47%. Hi-C scaffolding anchored 978.89 Mb (86.85% of the assembly) onto eight chromosome-level scaffolds. BUSCO analysis using the mollusca_odb10 dataset recovered 86.9% complete orthologs from the genome assembly. Repeat annotation identified 378.75 Mb of repetitive sequences (33.60% of the genome), with unclassified repeats and LTR elements as the dominant components. Gene annotation predicted 22,232 protein-coding genes, 209 tRNAs, 72 rRNAs, 88 snRNAs, and 10 snoRNAs. Functional annotation assigned database support to 155,611 predicted proteins or isoforms, corresponding to 98% of the total protein set. CAZy annotation identified 5371 carbohydrate-active enzyme entries, suggesting broad carbohydrate-processing potential. This genome provides a reference resource for comparative genomics, chromosome evolution, repeat dynamics, gene-family evolution, and freshwater adaptation studies in Viviparidae.

## Simple Summary

Freshwater snails are important members of aquatic ecosystems, but genomic information is still limited for many species. *Sinotaia angularis* is a viviparid snail with ecological and evolutionary relevance, yet no chromosome-level genome had previously been available for this species. In this study, we generated a 1.127 Gb genome assembly using long-read sequencing and Hi-C scaffolding. Most of the genome was organized into eight chromosome-level scaffolds, and 22,232 protein-coding genes were predicted. We also identified repetitive sequences, non-coding RNAs, and gene groups related to general biological functions such as metabolism, cellular regulation, and carbohydrate processing. This genome provides a useful reference for future studies on freshwater snail biology, genome evolution, species identification, and adaptation to changing aquatic environments.

## 1. Introduction

Freshwater gastropods are important components of aquatic ecosystems, contributing to nutrient cycling, organic-matter processing, and food-web interactions [1,2,3]. Among them, members of the family Viviparidae, commonly known as river snails or viviparid snails, are widely distributed freshwater caenogastropods with broad ecological and evolutionary significance, including roles in benthic organic-matter processing and filter-feeding [4,5]. Viviparids are biologically distinctive because many species are ovoviviparous, retaining developing embryos within the female reproductive tract before releasing juvenile snails [5,6,7]. This reproductive strategy shapes population dynamics, juvenile survival, and responses to variable freshwater environments [6,8]. Studies on *Viviparus* species have shown that reproduction, fecundity, sex ratio, and population structure vary among habitat types and seasons, indicating that viviparid life histories are closely tied to local environmental conditions [6,7,8]. As freshwater habitats face increasing pressures from eutrophication, pollution, hydrological alteration, habitat degradation, and climate warming, genomic resources for viviparid snails are essential for understanding how these animals adapt to environmental stress and sustain viable populations [9,10].

Despite their ecological relevance, viviparid snails remain underrepresented in genomic studies relative to several other molluscan groups. Molluscan genomics has expanded considerably over the past decade, driven by assemblies of oysters, limpets, sea slugs, apple snails, freshwater pulmonate snails, and other gastropods [11,12,13,14,15,16]. These studies revealed that molluscan genomes are highly variable in size, repeat content, chromosome organization, and gene-family composition [11,14,15,16]. The Pacific oyster genome uncovered complex gene repertoires associated with stress response, shell formation, and sessile adaptation [11]. The genome of the schistosomiasis vector *Biomphalaria glabrata* (Say, 1818) provided a key resource for studying host–parasite interactions and immune-related genes in freshwater snails [12]. The chromosome-level genome of the golden apple snail, *Pomacea canaliculata* (Lamarck, 1822), offered insight into stress tolerance, invasive adaptation, and ecological plasticity [13], while comparative ampullariid genomes further clarified genomic features associated with divergence, invasiveness, and the repeated transition toward more terrestrial reproductive strategies [14]. More recently, chromosome-scale ampullariid genomes have enabled refined comparative analyses of genome evolution, ecological divergence, and environmental adaptation in apple snails [17]. Nonetheless, the number of high-quality genomes from Viviparidae remains limited, constraining comparative work within this family.

Recent assemblies have begun to close this gap. The chromosome-level genome of *Bellamya purificata* (Heude, 1890), currently treated as *Sinotaia quadrata* (W. H. Benson, 1842), provided insight into freshwater snail genome organization and established a reference for population and evolutionary studies [10]. A chromosome-level assembly of *Cipangopaludina cathayensis* (Heude, 1890), currently treated as *Margarya catayensis* (Heude, 1890), further expanded genomic resources for Viviparidae and indicated that freshwater viviparid genomes may show substantial variation in genome size, chromosome-scale organization, and gene-family evolution [18]. Genomic and transcriptomic work on *Bellamya aeruginosa* (Reeve, 1863), a name also associated with the *S. quadrata* taxonomic complex, has additionally linked viviparid genomic data with environmental stress responses, including responses to wastewater exposure and temperature stress [19]. These studies underscore the value of viviparid reference genomes while also highlighting the need for additional assemblies from diverse taxa to clarify which genomic features are conserved across the family and which are lineage-specific.

**Figure 1:**
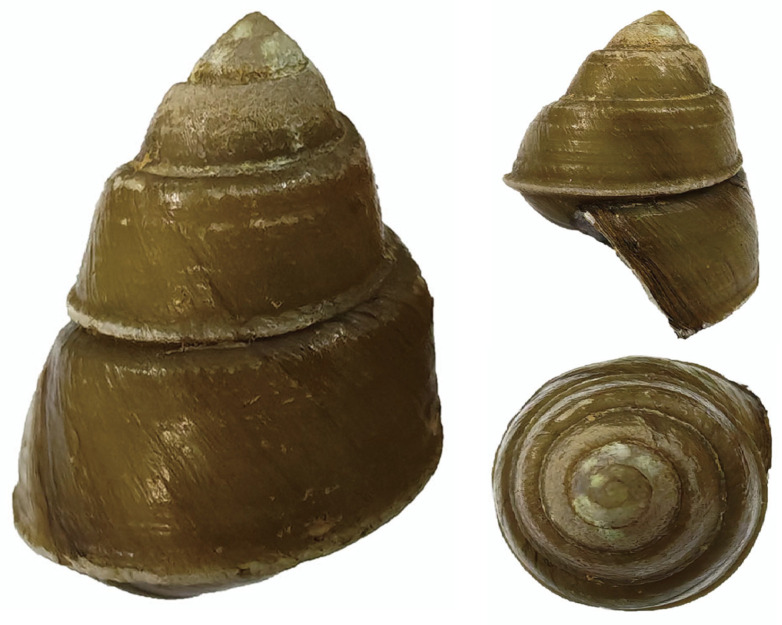
The morphological view of *S. angularis*.

*Sinotaia angularis* is a freshwater viviparid snail (Figure 1), yet genomic resources for this species have been entirely lacking. This represents a significant limitation because species and varieties within Viviparidae can be difficult to distinguish based only on morphology. Shell form, reproductive anatomy, opercular features, and radular characters are commonly used in viviparid taxonomy, but these traits may show intraspecific variation, convergence, or limited diagnostic resolution [5,20]. Molecular studies have further shown that phylogenetic relationships within Viviparidae may be complex, including cases of paraphyly and discordance among traditional taxonomic groupings [20,21]. In this context, genome-scale resources can provide an additional source of evidence for future studies of phylogenetic placement, population structure, genetic diversity, and evolutionary history in viviparid snails [22,23].

High-quality genome assemblies are particularly informative for addressing several outstanding questions in *S. angularis*. First, a chromosome-level assembly can reveal whether its genome organization is similar to other viviparids such as *Sinotaia* and *Margarya*, or whether it has experienced lineage-specific chromosomal rearrangements [10,18]. Second, repeat annotation can help explain genome-size evolution, given that transposable elements and unclassified repeats are major drivers of genome expansion in gastropods and other molluscs [24,25]. Third, protein-coding gene prediction and functional annotation can provide a framework for exploring putative genes and pathways associated with reproduction, development, environmental response, immunity, metabolism, mucus production, and shell-associated processes [10,11,13,19]. Finally, a reference genome provides the foundation for future comparative genomics, transcriptomics, population genomics, and conservation-oriented studies in Viviparidae [10,15,18,19].

In this study, we generated a chromosome-level genome assembly for *S. angularis* using PacBio HiFi long-read sequencing combined with Hi-C scaffolding. We evaluated genome continuity and completeness, characterized the repetitive element landscape, predicted protein-coding and non-coding genes, and functionally annotated the gene set using multiple public databases. Comparison of the resulting genomic features with published molluscan and viviparid genomes positions this assembly within a broader evolutionary context and provides a reference for future studies of genome evolution, genetic diversity, and population-level variation in viviparid snails.

## 2. Materials and Methods

### 2.1. Sample Preparation and Genomic DNA Quality Control

A total of 20 adult *S. angularis* specimens were collected in 2024 from Honghu City, Hubei Province, China. The wild founder population was originally established in 2020 and maintained under four generations of continuous selective breeding, resulting in a representative F4-generation laboratory lineage used in this study. Ten individuals were used for whole-genome sequencing and genomic analysis, while the remaining ten were retained as shell reference specimens at the Liuzhou Aquaculture Technology Extending Station under specimen code YYZ-RJ20240410-001.

High-molecular-weight (HMW) genomic DNA was extracted and prepared for both PacBio HiFi sequencing and Hi-C library construction. Because long-read sequencing and chromosome conformation capture both require high-integrity, high-purity DNA, extracted material was subjected to multiple quality-control steps prior to library preparation. DNA purity was assessed using a NanoDrop spectrophotometer (NanoDrop One, Thermo Fisher Scientific, Waltham, MA, USA) by measuring OD260/280 and OD260/230 absorbance ratios; acceptable thresholds were OD260/280 ≥ 1.8 and OD260/230 ≥ 1.5, confirming the absence of protein, phenol, and solvent contamination. DNA concentration was independently quantified using a Qubit 3.0 fluorometer (Thermo Fisher Scientific), and Qubit readings were compared with NanoDrop measurements to detect any co-purified non-nucleic acid contaminants. DNA integrity was confirmed by agarose gel electrophoresis to verify that the HMW genomic DNA was sufficiently intact for long-read library construction.

### 2.2. Molecular Species Identification and Phylogenetic Analysis

To provide molecular support for species identification, mitochondrial 16S rRNA and cytochrome c oxidase subunit I (COI, cox1) sequences obtained from the sequenced specimen were used for preliminary screening against the NCBI nucleotide database using BLASTn https://blast.ncbi.nlm.nih.gov/Blast.cgi (accessed on 20 June 2026). Reference sequences were selected based on sequence identity, query coverage, E-value, and taxonomic relevance within Viviparidae. The selected sequences, together with the sequence generated in this study and *M. catayensis* (NC_025577.1) as the outgroup, were aligned using ClustalW in MEGA 6.0. Phylogenetic relationships were inferred using the Maximum Likelihood method with the default substitution model in MEGA 6.0 and 1000 bootstrap replicates. Species names were verified and updated according to the currently accepted taxonomy in MolluscaBase where relevant.

### 2.3. PacBio HiFi Library Construction and Sequencing

PacBio HiFi (high-fidelity) sequencing was performed to generate long, highly accurate reads for de novo genome assembly [26]. Following DNA quality control, HMW genomic DNA was mechanically sheared to a target insert size of approximately 10 kb using a g-TUBE device (Covaris, Woburn, MA, USA). Sheared DNA was treated with exonuclease VII to remove 3′ single-stranded overhangs, after which damage repair was performed to correct single-strand nicks, missing bases, and oxidative lesions. The resulting fragments were end-repaired to produce blunt ends and ligated to barcode-containing SMRTbell dumbbell adapters. Exonuclease digestion was applied to remove unligated or incorrectly adapted molecules. The SMRTbell library was purified using 0.45× PB magnetic beads (Pacific Biosciences, Menlo Park, CA, USA) to enrich appropriately sized fragments and remove short-fragment contaminants. Library quality was verified by Qubit 3.0 fluorometry for concentration measurement and by an Agilent 2100 Bioanalyzer (Agilent Technologies, Santa Clara, CA, USA) for insert-size distribution; only libraries meeting the target concentration and size criteria proceeded to sequencing. Sequencing was performed on the PacBio Revio platform (Pacific Biosciences) in CCS (HiFi) mode using Revio Binding Kit 2.0, Revio Sequencing Kit 2.1, and Revio SMRT Cell 1M v2 (Pacific Biosciences). Raw CCS data were processed using SMRT Link v13.0 (Pacific Biosciences) to generate HiFi reads for downstream assembly.

### 2.4. Hi-C Library Construction and Sequencing

Hi-C sequencing was performed to obtain chromatin interaction data for chromosome-level scaffolding [27]. The Hi-C protocol exploits the principle that DNA fragments in close three-dimensional proximity within the nucleus are preferentially cross-linked and co-ligated, particularly fragments residing on the same chromosome. Briefly, tissue was fixed with 1% paraformaldehyde to preserve native chromatin conformation. After cell lysis, cross-linked chromatin was digested with the restriction enzyme MboI to generate cohesive ends. Restriction fragment ends were repaired and filled in with biotinylated nucleotides to mark ligation junctions. Proximity ligation was performed using T4 DNA ligase, after which cross-links were reversed by proteinase K digestion and DNA was purified. The purified DNA was sonicated to fragments of approximately 300–500 bp, and biotin-marked ligation products were enriched using streptavidin-coated magnetic beads. Captured fragments were used for Illumina paired-end library preparation, comprising end repair, A-tailing, adapter ligation, PCR amplification cycle estimation, and size-selection purification. Library concentration was initially assessed using a Qubit 2.0 fluorometer (Thermo Fisher Scientific) after dilution to 1 ng/μL. Insert-size distribution was verified using an Agilent 2100 Bioanalyzer (Agilent Technologies). Effective library concentration was determined by quantitative PCR; libraries with an effective molarity exceeding 2 nM were considered suitable for sequencing. Qualified Hi-C libraries were pooled according to effective concentration and target data volume, then sequenced on an Illumina HiSeq platform (Illumina, San Diego, CA, USA) to generate paired-end reads.

### 2.5. PacBio HiFi Read Statistics

PacBio HiFi reads were summarized prior to assembly to evaluate sequencing output. Metrics calculated included total read count, total bases, read N50, read N90, mean read length, median read length, maximum read length, and minimum read length. Read length distribution was visualized to confirm suitability for long-read de novo assembly. Genome coverage was estimated by dividing total HiFi bases by the final genome assembly size.

### 2.6. De Novo Genome Assembly

HiFi reads were assembled de novo using Hifiasm [28]. Hifiasm was selected because it is specifically designed for highly accurate long reads and constructs haplotype-resolved assemblies using phased assembly graphs. Assembly was run with default parameters to produce the primary contig assembly, which served as the basis for downstream Hi-C scaffolding. Assembly statistics were calculated for both the contig-level and final scaffold-level assemblies, including total length, sequence count, N50, L50, N90, L90, mean sequence length, median sequence length, maximum sequence length, minimum sequence length, and GC content. N50 was defined as the sequence length at which 50% of the total assembled bases are contained in sequences of at least that length; L50 was the minimum number of sequences required to reach that threshold. N90 and L90 were defined analogously for 90% of the assembly.

### 2.7. Hi-C Read Filtering and Quality Control

Raw Hi-C reads were filtered and processed prior to chromosomal scaffolding. Adapter trimming and quality filtering were first performed using fastp [29]; adapter sequences were removed, low-quality bases were trimmed, and reads shorter than 50 bp post-trimming were discarded. Bases in low-quality runs with Phred scores below 20 were also trimmed. Filtered reads were then subjected to Hi-C-specific quality control using HiCUP [30], which called Bowtie2 [31] to align reads to the genome assembly, splitting reads spanning restriction enzyme ligation junctions before alignment. Only paired-end reads with both mates uniquely mapping to the genome were retained. Invalid Hi-C read pairs were removed, including pairs located more than 500 bp from restriction sites, same-fragment circularized reads, dangling-end reads, same-fragment internal reads, re-ligation products, contiguous sequence artifacts, wrong-size fragments, and PCR duplicates. The effective Hi-C data rate was defined as the proportion of valid read pairs retained after adapter trimming, genome mapping, restriction-site filtering, removal of invalid ligation products, and duplicate removal.

### 2.8. Chromosome-Level Genome Scaffolding

Valid Hi-C read pairs were used for chromosome-level scaffolding on two guiding principles: (1) interaction frequency is higher between sequences on the same chromosome than between those on different chromosomes; and (2) within a chromosome, interaction frequency decreases with increasing linear genomic distance. Valid Hi-C interaction data produced by HiCUP were used as input for Juicer [32] to construct a genome-wide interaction matrix, which was then used by 3D-DNA [33] to cluster, order, and orient contigs into chromosome-level scaffolds. The resulting assembly was inspected and manually corrected using Juicebox Assembly Tools (JBAT) [34], with corrections focused on misjoins, incorrect orientations, and ordering errors identified from the Hi-C contact map. The corrected assembly was retained as the final chromosome-level reference. The chromosome anchoring rate was calculated as the total length of chromosome-level scaffolds divided by the total assembly length. A Hi-C heatmap was generated to assess scaffolding quality; strong intra-chromosomal interaction signals along the diagonal were interpreted as evidence of accurate chromosome reconstruction.

### 2.9. Telomeric Repeat Detection

Telomeric repeat sequences were searched at both termini of all chromosome-level scaffolds to assess chromosome-end completeness. The canonical metazoan telomeric hexamer TTAGGG (5′-TTTAGGG-3′ forward strand; complementary strand 5′-CCCTAAA-3′) was used as the query motif. Candidate telomeric signals were recorded for each chromosome, and results were tabulated separately for 5′ and 3′ ends. Absence of a detected signal was interpreted with caution and was not taken as evidence that telomeric sequences are biologically absent, given that assembly continuity at chromosome termini may be imperfect.

### 2.10. Genome Completeness Assessment

Genome assembly completeness was evaluated using BUSCO [35,36]. The assembly was assessed in genome mode using BUSCO v5 with the mollusca_odb10 lineage dataset (5295 conserved molluscan ortholog groups). BUSCO output was categorized as complete single-copy, complete duplicated, fragmented, or missing. The percentage of complete BUSCOs served as the primary measure of conserved gene-space completeness. The predicted protein set was additionally assessed in protein mode using the same mollusca_odb10 dataset; because multiple isoforms per locus were retained, elevated duplicated BUSCO counts in protein mode were interpreted with caution.

### 2.11. Read Mapping and Genome Coverage Evaluation

To evaluate assembly support, PacBio HiFi reads were aligned back to the final genome assembly using minimap2 v2.28 [37]. Average read depth and coverage breadth were calculated for each chromosome-level scaffold. Genome-wide read-depth distribution was visualized to confirm uniform coverage. Regions of unusually high depth were flagged as potentially representing collapsed repeats, segmental duplications, or assembly artifacts.

### 2.12. Repeat Annotation

Repetitive elements were annotated using a combination of de novo and homology-based approaches to maximize detection of both known and lineage-specific repeats. Tandem repeats were identified using Tandem Repeats Finder v4.09 [38]. Microsatellite sequences (simple sequence repeats) were predicted using MISA [39]. LTR retrotransposons were identified de novo using LTR_FINDER [40] and LTRharvest [41]; results from both tools were integrated using LTR_retriever v2.7 [42] to produce a refined, non-redundant LTR annotation set. A species-specific de novo repeat library was built using RepeatModeler2 [43] https://www.repeatmasker.org/dev/RepeatModeler/ (5 December 2024), which employs RECON, RepeatScout, and structural LTR detection to identify and classify consensus repeat families. This library was combined with known repeat databases and used as input for RepeatMasker v4.1.6 [44] https://www.repeatmasker.org/ (6 December 2024) to annotate LINEs, SINEs, LTR elements, DNA transposons, unclassified repeats, simple repeats, and low-complexity regions across the genome. Annotation results were summarized by repeat class, element count, total occupied length, and proportion of the genome.

### 2.13. Non-Coding RNA Annotation

Non-coding RNA (ncRNA) genes were predicted from the final genome assembly. Transfer RNA genes were identified using tRNAscan-SE v1.3.1 [45] with default eukaryotic parameters. Ribosomal RNA genes were predicted using RNAmmer v1.2 [46] with eukaryotic models covering large-subunit, small-subunit, and 5S/5.8S/8S rRNA. Small nuclear RNA and small nucleolar RNA genes were identified using cmscan v1.1.2 [47] against the Rfam v14.0 database [48]. For each ncRNA class, the number of predicted genes, total length, and average length were tabulated. rRNA subclasses were summarized separately to distinguish 18S, 28S, 5.8S, 5S, and 8S rRNA annotations.

### 2.14. Protein-Coding Gene Prediction

Protein-coding genes were predicted using an integrated, evidence-based annotation strategy. Prior to gene prediction, repetitive regions were soft-masked using RepeatMasker to reduce spurious predictions arising from transposable elements. The pipeline incorporated ab initio prediction, homology-based prediction, and transcript-guided evidence. MAKER v2.31.10 [49] was used to integrate homologous protein sequences and transcript evidence into initial gene models, which were then used to train AUGUSTUS v3.3.3 [50] for ab initio prediction. Where RNA-seq data were available, trimmed reads were aligned to the genome using HISAT2 v2.0.0 [51]; genome-guided transcript assembly was performed using Trinity v2.3.2 [52], and open reading frames were identified using TransDecoder v2.01 [53]. Evidence from ab initio predictions, homology alignments, and transcript assemblies was integrated into a final non-redundant gene model set using EvidenceModeler v1.1.1 [53] (https://github.com/TransDecoder/TransDecoder (accessed on 20 June 2026)). The final annotation was summarized by gene count, total and average gene length, protein isoform count, total and average protein length, and CDS GC ratio. Protein counts were interpreted as isoform-level figures, as multiple isoforms may be predicted per gene locus.

### 2.15. Functional Annotation of Protein-Coding Genes

Predicted protein sequences were functionally annotated by similarity searches against multiple public databases. BLASTP v2.6.0+ [54] was used to query proteins against the NCBI non-redundant (NR) protein database, Swiss-Prot, TrEMBL, and KOG databases, applying an E-value threshold of 1 × 10^−5^. The best-supported hit was retained for each protein. KEGG annotation [55] assigned predicted proteins to biological pathways and functional categories. Pfam domain searches were conducted using PfamScan v1.6 [56] against the Pfam database. Gene Ontology (GO) terms were assigned based on NR and database-derived annotations. Annotation coverage for each database was calculated as the proportion of predicted protein entries receiving at least one match. GO results were classified into biological process, molecular function, and cellular component categories. KEGG results were grouped into pathway categories encompassing cellular processes, environmental information processing, genetic information processing, metabolism, and organismal systems. KOG results were grouped into eukaryotic orthologous functional categories.

### 2.16. Species Distribution of NR Hits

To evaluate the taxonomic profile of protein homology matches, best-hit species assignments from NR BLASTP searches were tallied. The most frequently represented taxa were used to assess whether predicted proteins showed the expected similarity to molluscan and gastropod sequences, and to flag potential contamination if non-metazoan taxa were disproportionately represented.

### 2.17. Specialized Functional Annotation

Additional functional annotations were performed against four specialist databases: CAZy [57], CARD [58], PHI-base [59], and VFDB [60]. CAZy was used to identify carbohydrate-active enzymes (CAZymes), classified into glycoside hydrolases (GHs), glycosyltransferases (GTs), auxiliary activity enzymes (AAs), carbohydrate-binding modules (CBMs), carbohydrate esterases (CEs), and polysaccharide lyases (PLs). CARD identified predicted proteins similar to characterized antimicrobial resistance determinants; PHI-base identified homologs of experimentally validated pathogen-host interaction genes; and VFDB detected homologs of known bacterial virulence factor-associated proteins. All specialized database annotations were interpreted strictly as sequence-similarity-based information and were not used to infer pathogenicity, virulence, or antimicrobial resistance in *S. angularis*.

### 2.18. Genome-Wide Visualization

Genome-wide distribution of annotated genomic features was visualized using Circos [61]. The circos plot displayed the eight chromosome-level scaffolds with five concentric tracks from outermost to innermost: gene density, transposon density, repeat sequence density, and GC content (all calculated in 200 kb sliding windows, with GC content windows above and below the genome-wide mean shown in red and green respectively), followed by intra-genomic synteny blocks identified using MCScanX based on predicted protein sequences. This integrated visualization provided a genome-scale overview of the distribution of coding, repetitive, and structural features across the chromosome-level assembly.

### 2.19. Data Processing and Summary Statistics

All summary statistics reported in this study were derived from the assembled genome, annotation output files, and quality assessment results. Sequencing statistics were calculated from HiFi read data. Assembly statistics were computed separately for the contig assembly, final scaffold assembly, chromosome-level scaffolds, and unanchored sequences. Functional annotation summaries were tabulated from database search output files. Repeat, ncRNA, BUSCO, and specialized database results were compiled from their respective tool outputs.

## 3. Results

### 3.1. Molecular Species Identification and Phylogenetic Placement

**Figure 2:**
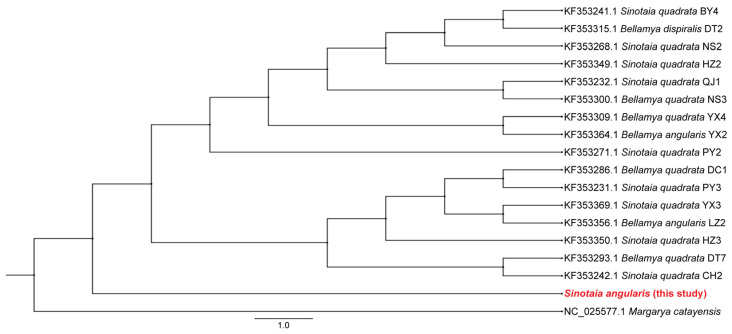
Maximum Likelihood phylogenetic tree based on mitochondrial 16S rRNA sequences. *M. catayensis* (NC_025577.1; originally deposited as *C. cathayensis*) was used as the outgroup, and the sequence generated in this study is highlighted in red. Species names were updated according to currently accepted taxonomy where reliable names were available; otherwise, original NCBI record names were retained.

Mitochondrial 16S rRNA and COI (cox1) sequences from the sequenced specimen were used for preliminary screening against the NCBI nucleotide database using BLASTn. Based on sequence similarity and taxonomic relevance, closely related reference sequences were selected for phylogenetic analysis. The Maximum Likelihood phylogenetic tree was constructed based on the 16S rRNA alignment. The sequence generated in this study clustered within the *Sinotaia* lineage and was distinctly separated from the outgroup *M. catayensis* (Figure 2). This phylogenetic placement supports the assignment of the sequenced specimen to *S*. *angularis* and is consistent with its current taxonomic classification.

### 3.2. Sequencing Output and Read Characteristics

To generate a high-quality reference genome for *S. angularis*, PacBio HiFi long-read sequencing was performed. A total of 4,836,738 HiFi reads were obtained, producing 74.08 Gb of sequencing data. The read N50 was 15.53 kb, the read N90 was 10.90 kb, the mean read length was 15.32 kb, and the median read length was 14.02 kb; the longest individual read reached 63.37 kb (Figure S1). Based on the final genome assembly size of 1.127 Gb, the HiFi data provided approximately 65.7-fold genome coverage. This sequencing depth provided sufficient information for resolving long genomic regions, repetitive sequences, and complex genome structures. Sequencing statistics are summarized in Table 1.

### 3.3. Genome Assembly Statistics

PacBio HiFi reads were assembled de novo using Hifiasm. The initial contig-level assembly spanned 1.126 Gb across 9252 contigs, with a contig N50 of 3.10 Mb and a contig N90 of 40.04 kb. The longest contig reached 18.97 Mb and the mean contig length was 121.67 kb. The GC content of the contig assembly was 34.51%. These results indicate that the long-read assembly produced a relatively continuous genome, with multiple megabase-scale contigs before chromosome-level scaffolding. The GC content was consistent across the final assembly and chromosome-level sequences, suggesting no strong GC bias in the assembled genome. Assembly statistics are summarized in Table 2.

### 3.4. Hi-C-Assisted Chromosome-Level Assembly

**Figure 3:**
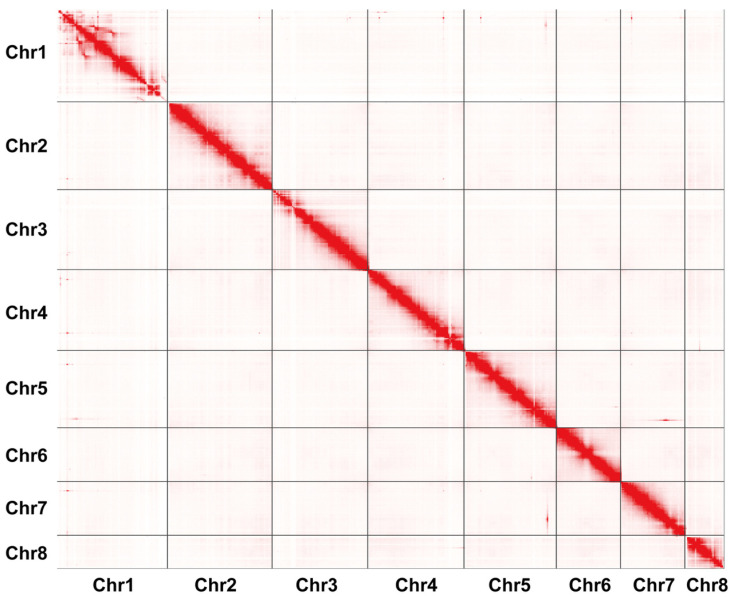
The Hi-C interaction heatmap of *S. angularis*.

Hi-C data were used to anchor, order, and orient contigs into chromosome-level scaffolds. The resulting final genome assembly comprised 6276 sequences with a total length of 1.127 Gb and an overall GC content of 34.47% (Table 3). High assembly continuity was demonstrated by a scaffold N50 of 141.87 Mb and an L50 of 3, indicating that just three scaffolds accounted for fully half of the assembled genome length, while the scaffold N90 reached 91.01 kb. In total, eight chromosome-level scaffolds were successfully reconstructed, ranging in size from 58.05 Mb to 163.88 Mb (Figure 3). Together, these pseudochromosomes spanned 978.89 Mb, successfully anchoring 86.85% of the assembly (Table S1). The remaining unanchored sequences, designated as ChrUN, totaled 148.28 Mb (13.15% of the assembly). The Hi-C contact map showed clear intra-chromosomal interaction signals along the diagonal of each chromosome, supporting the overall chromosome-scale organization of the assembly.

### 3.5. Assembly Completeness and Read Support

Genome assembly completeness was evaluated using BUSCO v5 with the mollusca_odb10 dataset (5295 orthologs). A total of 4599 complete BUSCO genes were identified, corresponding to 86.9% completeness; of these, 4538 were single-copy and 61 were duplicated. An additional 51 BUSCOs were fragmented and 645 were missing (Table S2; Figure S3). HiFi read mapping confirmed 100% coverage across all eight chromosomes, with per-chromosome sequencing depths ranging from 45-fold to 63-fold (Table S3). Telomeric repeat motifs were detected at the 3′ end of Chr3; while no terminal telomeric signals were detected at the remaining chromosome ends (Table S4), which may reflect incomplete recovery of chromosome termini in the current assembly.

### 3.6. Genome Repeat Landscape

A total of 378.75 Mb of repetitive sequence was identified, accounting for 33.60% of the assembled genome. Interspersed repeats were the dominant class, covering 340.52 Mb (30.21% of the genome). Unclassified repeats formed the largest single category at 215.32 Mb (19.10%). Among classified transposable elements, LTR retrotransposons were the most abundant at 75.20 Mb (6.67%), followed by LINEs at 27.71 Mb (2.46%) and DNA transposons at 22.28 Mb (1.98%). Simple repeats accounted for 37.19 Mb (3.30%) and low-complexity regions for 3.53 Mb (0.31%). No SINE elements were detected. Repeat annotation results are summarized in Table S5.

### 3.7. Protein-Coding Gene Features

Protein-coding gene annotation identified 22,232 genes in the *S. angularis* genome (Table 4). Annotated gene regions spanned 670.50 Mb in total, with an average gene length of 30.16 kb. The predicted CDS GC ratio was 44.78%, which was higher than the overall genomic GC content of approximately 34.5%, reflecting the expected compositional difference between coding and non-coding regions. A total of 157,968 protein isoforms were reported, with a combined length of 61.88 million amino acids and an average predicted protein length of 391.7 amino acids. The large difference between the number of genes and protein isoforms suggests that multiple transcript or protein isoforms were predicted for many loci.

### 3.8. Non-Coding RNA Repertoire

Non-coding RNA annotation predicted 209 tRNA genes with a combined length of 15.60 kb and a mean length of 74 bp (Table 5). An additional 88 snRNA genes and 10 snoRNA genes were detected, with mean lengths of 126.5 bp and 171 bp, respectively (Table 5). Ribosomal RNA annotation identified 72 rRNA genes in total, comprising 1 18S rRNA, 2 28S rRNAs, 4 5.8S rRNAs, 32 5S rRNAs, and 33 8S rRNAs (Table 6). 

### 3.9. Functional Annotation of Predicted Proteins

**Figure 4:**
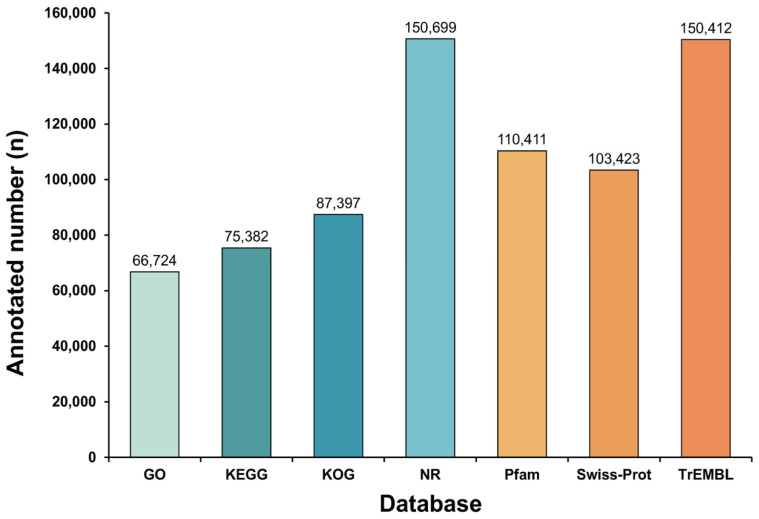
The summary of functional annotation across public databases.

Functional annotation was performed against NR, TrEMBL, Swiss-Prot, Pfam, KOG, KEGG, and GO databases. A total of 155,611 predicted proteins or isoforms received at least one database annotation, corresponding to 98% of the full protein set. NR and TrEMBL each annotated approximately 95% of the protein set (150,699 and 150,412 entries respectively). Swiss-Prot annotated 103,423 entries (65%), Pfam annotated 110,411 entries (69%), KOG annotated 87,397 entries (55%), KEGG annotated 75,382 entries (47%), and GO annotated 66,724 entries (42%). Annotation statistics are summarized in Figure 4.

### 3.10. GO Functional Classification

**Figure 5:**
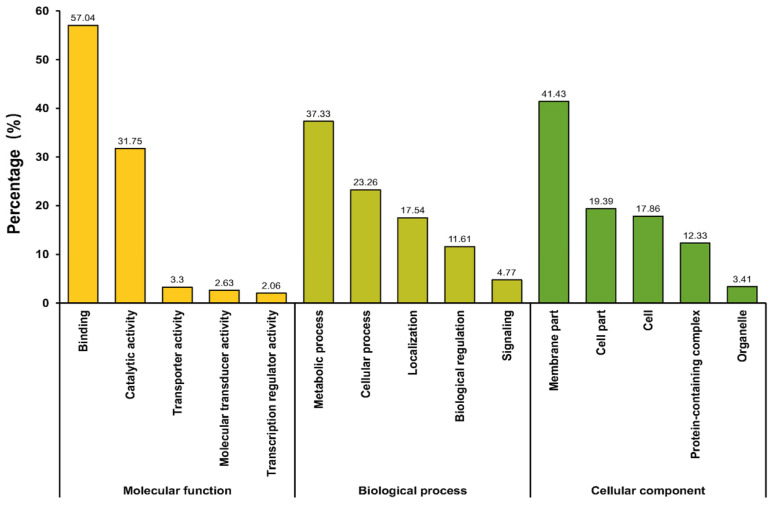
Major GO functional categories.

GO classification assigned predicted proteins to molecular function, biological process, and cellular component categories. Within molecular function, binding was the most abundant term (57.04%), followed by catalytic activity (31.75%). Within the biological process, the metabolic process was dominant (37.33%), followed by the cellular process (23.26%), localization (17.54%), and biological regulation (11.61%). Within the cellular component, the membrane part was the most represented term (41.43%) (Figure 5).

### 3.11. KEGG Pathway Annotation

**Figure 6:**
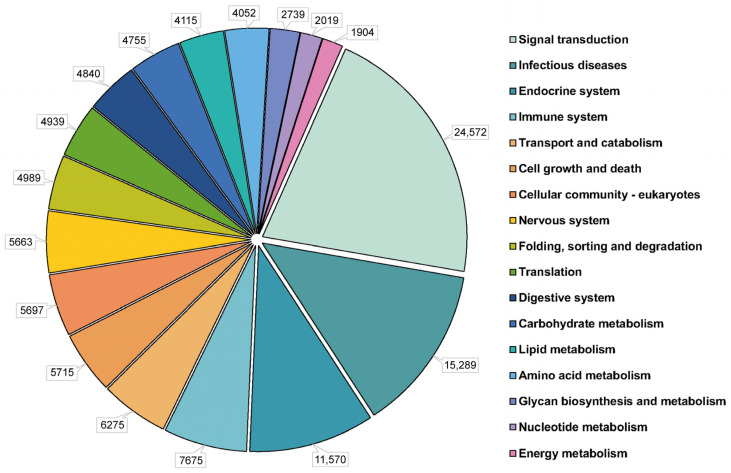
The representative KEGG pathway categories.

KEGG annotation assigned predicted proteins to a wide range of biological pathways. Signal transduction emerged as the largest KEGG category, with 24,572 annotations, suggesting an extensive repertoire of genes involved in environmental sensing, cellular communication, and regulatory responses. Metabolic pathways were also highly represented, including carbohydrate, amino acid, lipid, nucleotide, and energy metabolism, as well as glycan biosynthesis and metabolism (Figure 6). Crucially, while numerous sequences were mapped to KEGG disease-related pathways, such as infectious diseases and neurodegenerative diseases, these classifications are assigned based on sequence homology to established reference genes. These annotations do not imply active disease states within the organism; instead, they reflect conserved molecular pathways that are fundamental to animal and eukaryotic biology.

### 3.12. KOG Functional Classification

**Figure 7:**
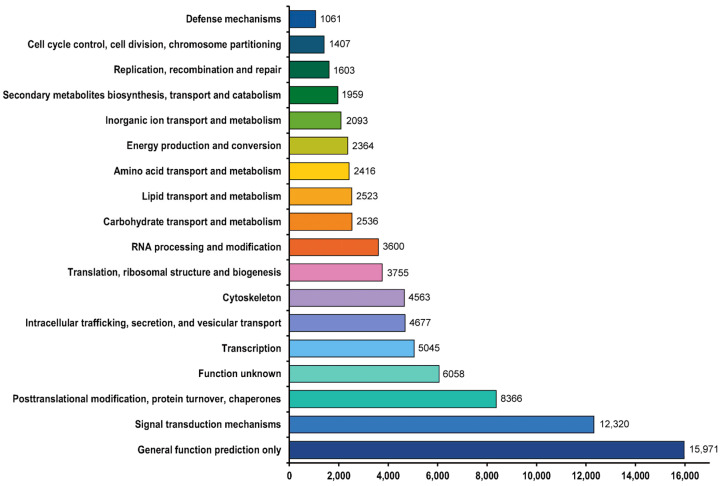
Major KOG functional categories.

KOG annotation assigned 87,397 predicted proteins to eukaryotic orthologous groups. The largest category was ‘general function prediction only’, with 15,971 annotations, indicating that many genes had conserved domains or homologs but lacked detailed functional characterization. Signal transduction mechanisms formed the second-largest category, with 12,320 annotations, consistent with the KEGG results showing abundant signaling-related genes (Figure 7).

### 3.13. Specialized Annotation Profile

Specialized annotation against CARD, CAZy, PHI-base, and VFDB databases identified 14,589 annotated entries in total, corresponding to 9% of the predicted protein set. PHI-base yielded the most matches (7562 entries; 4%), followed by CAZy (5371 entries; 3%), VFDB (2467 entries; 1%), and CARD (196 entries; <1%) (Figure S3). These specialized annotations warrant cautious interpretation. Matches to the PHI, VFDB, or CARD databases do not directly confirm active pathogenicity, virulence, or antimicrobial resistance phenotypes within the host snail. Rather, they reflect sequence homology to cataloged proteins, many of which likely represent deeply conserved, basal cellular processes shared across diverse evolutionary lineages.

### 3.14. Carbohydrate-Active Enzyme Repertoire

**Figure 8:**
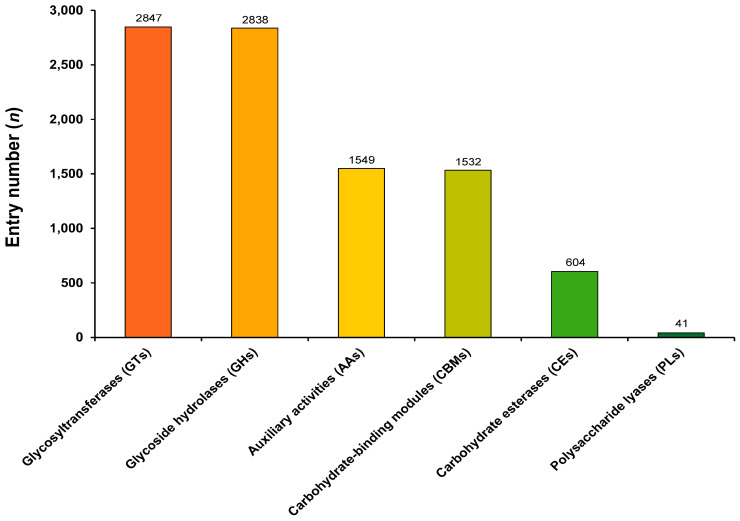
CAZy enzyme class distribution.

CAZy annotation identified 5371 carbohydrate-active enzyme entries distributed across six classes. Glycosyltransferases were the most abundant (2847 entries), followed by glycoside hydrolases (2838 entries), auxiliary activity enzymes (1549 entries), carbohydrate-binding modules (1532 entries), carbohydrate esterases (604 entries), and polysaccharide lyases (41 entries) (Figure 8). The abundance of glycosyltransferases and glycoside hydrolases suggests broad potential for carbohydrate synthesis, modification, and degradation. This enzymatic repertoire may be relevant to multiple physiological processes in *S. angularis*, including dietary digestion, extracellular matrix remodeling, glycan metabolism, and shell-associated biomineralization. The prevalent representation of carbohydrate-binding modules suggests that a substantial proportion of these predicted enzymes are optimized to interface with complex structural polysaccharides.

### 3.15. Genome Feature Visualization

Genome-wide feature distribution was visualized using Circos. The circos plot was used to summarize chromosome-level genome organization and the distribution of annotated genomic features across the eight chromosome-level scaffolds (Figure S4). The visualization confirmed that gene density and repeat density vary along individual chromosomes, with no obvious large-scale clustering of all features into a single chromosomal region, consistent with a relatively balanced genome organization.

### 3.16. Summary of Genome Features

The genome assembly of *S. angularis* produced a 1.127 Gb reference genome with a scaffold N50 of 141.87 Mb. Hi-C scaffolding anchored 978.89 Mb of the assembly onto eight chromosome-level scaffolds (86.85%), with the remaining sequences unanchored (Table 7). BUSCO analysis recovered 86.9% complete molluscan orthologs from the genome assembly and 88.6% from the predicted protein set. Repeat annotation identified 33.60% repetitive sequence, dominated by unclassified repeats and LTR elements. Gene prediction identified 22,232 protein-coding genes, 209 tRNAs, 72 rRNAs, 88 snRNAs, and 10 snoRNAs. Functional annotation yielded database support for 98% of the predicted proteins and isoforms, providing a useful basis for future investigations into viviparid genome evolution, environmental adaptation, gene family dynamics, and comparative molluscan genomics.

## 4. Discussion

The chromosome-level genome of *S. angularis* provides a new genomic resource for comparative studies of freshwater viviparid snails. Its value lies not only in the assembly statistics but also in its contribution to the growing comparative genomic framework of gastropods. Recent work has demonstrated substantial variation in genome size, repeat composition, chromosome-scale organization, and gene-space completeness across gastropod lineages, including freshwater Hygrophila (freshwater pulmonates), Architaenioglossa (e.g., Ampullariidae and Viviparidae), Stylommatophora (terrestrial snails), and marine caenogastropods [12,13,14,15,25,62]. The present assembly falls within the genome-size range reported for published viviparid genomes and, like several recent viviparid assemblies, was resolved into chromosome-scale scaffolds [10,18,19]. However, differences in chromosome numbers, repeat composition, and BUSCO gene-space completeness among these genomes indicate that broader comparative analyses are still needed. Together, these comparisons place the *S. angularis* assembly within the current genomic framework of Viviparidae and provide a basis for future comparative analyses. The 16S rRNA phylogenetic analysis, consistent with the BLAST-based preliminary screening and Maximum Likelihood reconstruction presented in the Results, supports the placement of the sequenced specimen within the *Sinotaia* lineage. However, broader taxon sampling and additional mitochondrial or nuclear markers will be needed to resolve species boundaries within closely related viviparid taxa.

A central feature of this assembly is its recovery of eight chromosome-level scaffolds. This is particularly relevant because several recently published viviparid genomes show a similar small-chromosome architecture. The genome published for *S*. *quadrata* was assembled at the chromosome level with eight pseudochromosomes [10]. A more recent *Sinotaia* assembly also reported a 1.2 Gb genome organized into eight pseudochromosomes and provided genomic evidence for stress-resistance mechanisms in this lineage [19]. The present genome of *S. angularis* is consistent with both *Sinotaia* references in chromosome number and assembly scale, supporting the possibility that eight chromosomes may reflect the true haploid complement of this lineage. Nevertheless, direct cytogenetic validation would be necessary before making a definitive karyotypic claim, as assembly based chromosome counts can occasionally reflect scaffolding artifacts or incomplete separation of closely related chromosomes [33].

This consistency becomes more striking when viewed against viviparids and non-viviparid gastropods that differ markedly in chromosome number. *M. catayensis*, another Viviparidae species, was assembled into nine pseudochromosomes with a 1.48 Gb genome and a scaffold N50 of 195.21 Mb [18]. Outside Viviparidae, chromosome counts can be considerably higher: *Achatina fulica* (Bowdich, 1822), currently treated as *Lissachatina fulica* (Bowdich, 1822), was anchored to 31 chromosomes [63], and *Rapana venosa* (Valenciennes, 1846) to 35 chromosomes in a 2.30 Gb assembly [62]. Future comparative synteny analyses among *S. angularis*, *S. quadrata*, *M. catayensis* and other viviparid assemblies will be needed to determine whether chromosome-number differences reflect chromosomal fusions, fissions, inversions, or assembly specific fragmentation [10,18,19].

At 1.127 Gb, the *S. angularis* genome falls within the size range reported for published viviparid genomes, including assemblies published for taxa currently treated within *Sinotaia* and *Margaya* [10,18,19]. It is smaller than the expanded genomes reported for more distantly related gastropods, such as *L*. *fulica* and *R*. *venosa* [62,63]. The repeat content provides a likely mechanistic explanation: repetitive sequences account for approximately one-third of the assembly, which is substantial but not extreme. By comparison, *R. venosa* has a repeat proportion of 57.72% [62], and *L. fulica* harbors very high repeat content contributing to its large genome size [63]. These comparisons suggest that *S. angularis* has not undergone the degree of repeat-driven expansion seen in some larger gastropod genomes.

The repeat landscape is nonetheless biologically informative. Unclassified repeats are the largest single category, followed by LTR elements, LINEs, and DNA transposons. The relatively high proportion of unclassified repeats in the *S. angularis* genome likely reflects the limited representation of mollusc- and lineage-specific repeat elements in current repeat databases [24,64]. Although these sequences were detectable as repetitive elements, their limited similarity to characterized repeat families prevented confident assignment to established repeat categories. Therefore, future de novo repeat-family reconstruction, repeat-age profiling using divergence estimates, and direct comparison with *Sinotaia* and *Margaya* repeat libraries would help clarify whether the repeat content of *S. angularis* reflects ancient accumulation or more recent transposable element activity [18,19,25].

The gene annotation is consistent with expectations for a viviparid snail. The predicted 22,232 protein-coding genes are comparable to the 22,702 reported for *M. catayensis* [18] and fall within the range documented for *S. quadrata* and other caenogastropods [10,62]. This high degree of consistency supports the biological plausibility of our annotation, indicating that the baseline gene set is not structurally inflated. However, the substantially larger volume of predicted protein isoforms warrants careful interpretation. The elevated proportion of duplicated BUSCOs observed within the total protein set likely captures alternative splicing events and redundant isoform models rather than true large-scale genomic duplications. Because the BUSCO metric evaluates completeness using conserved single-copy orthologs, apparent duplications in an unfiltered protein dataset must be interpreted cautiously when multiple isoforms are retained [35,65]. Consequently, to ensure precision in downstream comparative genomics, analyses must strictly utilize a filtered, longest-isoform protein set to prevent the artificial overestimation of gene-family sizes and orthogroup membership [66].

The BUSCO completeness profile warrants balanced interpretation. The genome assembly recovers most conserved molluscan orthologs, but its completeness is lower than some recent viviparid assemblies; *S. quadrata*, for example, recovered 97.80% of Mollusca BUSCO genes [19]. The lower BUSCO recovery observed here could reflect a combination of assembly gaps in gene-rich regions, annotation limitations, or divergence from the BUSCO reference set. It does not invalidate the assembly, which is strongly supported by full HiFi read coverage across all eight chromosomes, but it does suggest that transcriptome-guided annotation from multiple tissue types, particularly mantle, gonad, digestive gland, and embryonic tissue, would likely improve both gene completeness and isoform resolution [35,65,66].

Functional annotation highlights several biologically relevant gene categories, though these should be treated as working hypotheses rather than conclusions. Signal transduction, membrane-associated functions, transport, metabolic enzymes, and protein modification machinery are all prominently represented, which is broadly consistent with the functional repertoire expected for a freshwater gastropod occupying variable aquatic environments and must respond to changes in oxygen availability, temperature, pollutants, pathogens, and food resources [10,18,19]. In *S. quadrata*, transcriptomic responses to wastewater and thermal stress implicated lipid metabolism, antioxidant pathways, inflammatory signaling, and heat-shock proteins as key components of the stress response [19]. The present genome cannot by itself demonstrate similar adaptive mechanisms in *S. angularis*, but it provides the reference framework needed to test them.

The annotated CAZyme repertoire is one of the most promising parts of the genome for future biological interpretation. CAZy is a specialist database for carbohydrate-active enzymes that build, modify, and degrade complex carbohydrates and glycoconjugates [67]. The large numbers of glycosyltransferases, glycoside hydrolases, auxiliary activity enzymes, and carbohydrate-binding modules suggest a broad capacity for carbohydrate synthesis and degradation that may be relevant to multiple biological processes in *S. angularis*. In freshwater snails, carbohydrate-digesting enzymes have been implicated in the breakdown of starch, structural polysaccharides, and plant-derived substrates, and microbiome-associated CAZymes may contribute substantially to dietary processing [68,69]. Comparable investigations across the Gastropoda further reinforce the central importance of CAZymes and glycan metabolic pathways in systemic nutrient acquisition, mucosal biology, and extracellular matrix maintenance [13,70].

Beyond dietary digestion, these encoded enzymes are prime candidates for mediating mucosal secretion, epithelial surface interactions, extracellular matrix remodeling, and shell-associated glycan dynamics. Gastropod mucus is an intricate, glycoprotein-rich matrix essential for locomotion, adhesion, desiccation resistance, antimicrobial defense, and internal tissue lubrication, including the capture and transport of suspended particles during filter feeding. Furthermore, molluscan biomineralization studies have emphasized that shells are built through complex interactions between calcium carbonate and organic matrix components, including rapidly evolving secreted proteins, glycoproteins, polysaccharides, and chitin-associated molecules [71,72]. Consequently, while the CAZyme repertoire in *S. angularis* provides candidate enzymes that may be involved in digestive, extracellular and glycan-related functions, definitively linking specific genes to shell biomineralization will necessitate targeted, mantle-specific transcriptomic and proteomic validation [70,71,72].

The broader taxonomic context of *S. angularis* raises important evolutionary questions. Viviparidae is a morphologically and ecologically diverse family with substantial variation in shell form, anatomical traits, habitat use, and feeding strategies, as well as a wide geographic distribution and a long evolutionary history [5,22]. Recent comparative genomic work on *S. quadrata* suggested that gene-family expansions along the Viviparidae branch may be associated with morphogenesis and embryonic development, possibly in connection with viviparity [19]. As a member of the same family, the present *S. angularis* genome could be particularly useful for testing whether developmental gene-family patterns are shared across viviparids or restricted to specific lineages. This question is best addressed through rigorous orthology inference and formal gene-family expansion analysis rather than through functional annotation alone [73,74].

A final point concerns the interpretation of PHI-base, VFDB, CARD, and KEGG disease-related annotations. These databases are useful for identifying homologs of known stress-response, defense, transporter, and interaction-related proteins, but their labels can be misleading if read literally. PHI-base curates experimentally supported genes involved in pathogen-host interactions, VFDB focuses on bacterial virulence factors, and CARD provides curated information on antimicrobial resistance determinants [58,59,60]. Therefore, a match to any of these databases does not indicate that *S. angularis* is pathogenic, virulent, or antimicrobial resistant. Similarly, KEGG disease pathway categories are classification labels derived from conserved molecular interaction networks; they reflect pathway-level homology, not disease states in the snail [55,75]. The appropriate interpretation is that these annotations identify candidate proteins potentially involved in immunity, stress response, metabolism, transport, or cell signaling, hypotheses that can be tested with targeted experimental approaches.

Taken together, the genome of *S. angularis* represents a meaningful addition to the growing comparative genomic framework for Viviparidae. Its eight chromosome-level scaffolds are comparable to published *Sinotaia* assemblies, its genome size is intermediate among freshwater viviparids, and its repeat and gene content are consistent with related gastropods. The most productive next steps will be comparative synteny analysis with *S. quadrata* and *M. catayensis*; improved classification of unclassified repeats; construction of a non-redundant primary protein set; and tissue-specific transcriptomic validation of genes relevant to development, stress response, carbohydrate metabolism, and shell or mucus biology. With these additions, the genome of *S. angularis* could serve as a robust reference for studying chromosome evolution, freshwater adaptation, and functional diversification across Viviparidae.

## 5. Conclusions

This study presents a chromosome-level genome assembly of *S. angularis* constructed using PacBio HiFi long-read sequencing combined with Hi-C chromatin interaction scaffolding. The 1.127 Gb genome was anchored onto eight chromosome-level scaffolds and annotated with 22,232 protein-coding genes. Repeat and functional annotations revealed a moderately repetitive genome and broad gene repertoires related to metabolism and cellular regulation. Together, these findings position *S. angularis* within the genomic diversity of Viviparidae and provide a foundational resource for future comparative genomics, chromosome evolution studies, functional genomics, and freshwater adaptation research in this understudied snail family.

## Figures and Tables

**Table 1:** Summary of PacBio HiFi sequencing data.

Sample Parameters	HIFI Data
Reads number (*n*)	4,836,738
Total bases (bp)	74,078,394,585
Read N50 (bp)	15,528
Read N90 (bp)	10,896
Mean length (bp)	15,315
Median length (bp)	14,018
Maximum length (bp)	63,368
Minimum length (bp)	77

**Table 2:** Summary of contig-level genome assembly.

Assembly Version	Contig
Sequence number	9252
Total length (bp)	1,125,674,741
N50 (bp)	3,097,360
L50	89
N90 (bp)	40,036
L90	2305
Mean length (bp)	121,668
Median length (bp)	18,688
Maximum length (bp)	18,970,354
Minimum length (bp)	822
GC content (%)	34.51

**Table 3:** Summary of final assembly versions after Hi-C scaffolding.

Assembly Version	ALL	Chr	ChrUN
Sequence number (*n*)	6276	8	6268
Total length (bp)	1,127,162,741	978,885,730	148,277,011
N50 (bp)	141,867,416	141,867,416	39,973
L50	3	3	884
N90 (bp)	91,013	95,264,380	9550
L90	217	6	4146
Mean length (bp)	179,598	122,360,716	23,656
Median length (bp)	12,752	141,867,416	12,722
Maximum length (bp)	163,875,638	163,875,638	2,552,259
Total length (bp)	1,127,162,741	978,885,730	148,277,011

**Table 4:** Summary of protein-coding gene prediction.

Parameters	Descriptions
Gene number (*n*)	22,232
Total gene length (bp)	670,504,753
Average gene length (bp)	30,159.44
Protein isoform number (*n*)	157,968
Total protein length (aa)	61,878,207
Average protein length (aa)	391.71
CDS GC ratio (%)	44.78

**Table 5:** The summary of non-coding RNA annotation.

ncRNA Type	Number (*n*)	Total Length (bp)	Average Length (bp)
tRNA	209	15,597	74
rRNA	72	17,323	240.60
snRNA	88	11,131	126.49
snoRNA	10	1710	171

**Table 6:** The summary of rRNA subclasses.

rRNA Type	Number (*n*)	Total Length (bp)	Average Length (bp)
18S rRNA	1	1844	1844
28S rRNA	2	7443	3721.50
5.8S rRNA	4	573	143.25
5S rRNA	32	3741	116.91
8S rRNA	33	3722	112.79

**Table 7:** Key genome features of the snail species.

Feature	Value
Final assembly size	1,127,162,741 bp
Number of final assembly sequences	6276
Genome GC content	34.47%
Scaffold N50	141,867,416 bp
Chromosome-level scaffolds	8
Chromosome-anchored length	978,885,730 bp
Chromosome-anchored proportion	86.85%
Unanchored sequence length	148,277,011 bp
Genome BUSCO completeness	86.9%
Repeat content	378,751,486 bp
Repeat proportion	33.60%
Protein-coding genes	22,232
Protein isoforms	157,968
tRNAs	209
rRNAs	72
snRNAs	88
snoRNAs	10
Functionally annotated protein entries	155,611

## Data Availability

The raw sequencing data generated in this study, including PacBio HiFi genomic reads, Hi-C sequencing reads, Illumina RNA-seq reads, and PacBio Iso-Seq transcriptome reads, have been deposited in the NCBI Sequence Read Archive (SRA) under BioProject accession number PRJNA1473179. The chromosome-level genome assembly and annotation are available from the corresponding author upon reasonable request.

## Supplementary Materials

The following supporting information can be downloaded at: https://www.mdpi.com/article/10.3390/ani16131975/s1, Table S1: Chromosome-level scaffold statistics; Table S2: BUSCO completeness assessment of the genome assembly; Table S3: HiFi read depth and coverage across chromosome-level scaffolds; Table S4: Telomeric repeat detection in chromosome-level scaffolds; Table S5: Summary of repetitive sequence annotation; Figure S1. Length distribution of the PacBio HiFi sequencing reads used for the *S. angularis* genome assembly; Figure S2. BUSCO completeness assessment of the final *S. angularis* genome assembly using the mollusca_odb10 lineage dataset; Figure S3. Distribution of predicted proteins annotated across four specialized functional databases (PHI, CAZy, VFDB, and CARD); Figure S4. Circos plot illustrating the genome-wide landscape of genomic features across the eight chromosome-level scaffolds. Concentric tracks from outermost to innermost represent gene density, transposon density, repeat sequence density, GC content, and intra-genomic synteny blocks.
