# Supplementary material for: Chromosome-Level Genome Assembly and Annotation of the Freshwater Snail Sinotaia angularis (O. F. Müller, 1774)
Source: Animals (Basel). 2026 Jun 26;16(13):1975. doi: 10.3390/ani16131975 (PMC13359775; doi:10.3390/ani16131975)
Supplement: Supplementary file 1 [file animals-16-01975-s001.zip › Supplementary materials.pdf]

Table S1: Chromosome-level scaffold statistics

Chromosome	Length (bp)	Contig number (n)	Contig total length (bp)	GC content (%)
Chr1	163,875,638	1,195	163,278,638	34.42
Chr2	151,499,218	218	151,390,718	34.38
Chr3	141,867,416	483	141,626,416	34.49
Chr4	139,906,515	227	139,793,515	34.37
Chr5	135,087,578	255	134,960,578	34.36
Chr6	95,264,380	78	95,225,880	34.41
Chr7	93,337,329	108	93,283,829	34.57
Chr8	58,047,656	296	57,900,156	34.49
ChrAll	978,885,730	2,860	977,459,730	34.43

Table S2: BUSCO completeness assessment of the genome assembly

BUSCO category	Number (n)	Percentage (%)
Complete BUSCOs	4,599	86.9
Complete single-copy BUSCOs	4,538	85.7
Complete duplicated BUSCOs	61	1.2
Fragmented BUSCOs	51	1
Missing BUSCOs	645	12.1
Total BUSCO groups searched	5,295	100

Table S3: HiFi read depth and coverage across chromosome-level scaffolds

Chromosome	Length (bp)	Read depth (X)	Read coverage (%)
Chr1	163,875,638	45X	100
Chr2	151,499,218	62X	100

Chr3	141,867,416	54X	100
Chr4	139,906,515	60X	100
Chr5	135,087,578	59X	100
Chr6	95,264,380	63X	100
Chr7	93,337,329	60X	100
Chr8	58,047,656	51X	100

Table S4: Telomeric repeat detection in chromosome-level scaffolds.

Chromosome	5' telomere signal	3' telomere signal
Chr1	No	No
Chr2	No	No
Chr3	No	Yes: 141,861,659-141,867,416
Chr4	No	No
Chr5	No	No
Chr6	No	No
Chr7	No	No
Chr8	No	No

Table S5: Summary o�repetitive sequence annotation

Repeat category	Number of elements (n)	Length occupied (bp)	Percentage of genome (%)
Total masked repeats	NA	378,751,486	33.6
Total interspersed repeats	NA	340,523,588	30.21
SINEs	0	0	0
LINEs	57,246	27,713,613	2.46

LTR elements	117,122	75,204,404	6.67
DNA elements	42,939	22,282,347	1.98
Unclassified repeats	651,438	215,323,224	19.1
Simple repeats	562,461	37,188,118	3.3
Low-complexity regions	57,089	3,530,315 b	0.31

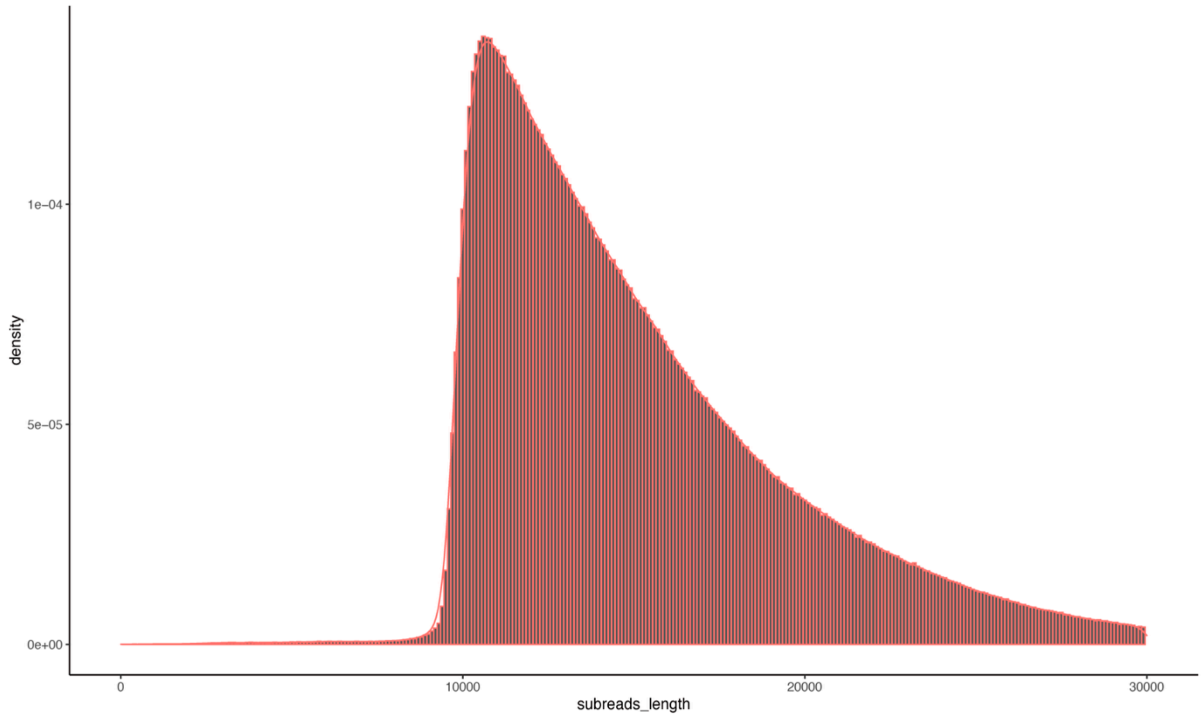

Figure S1. Length distribution o the PacBio HiFi sequencing reads used or the *Sinotaia angularis* genome assembly.

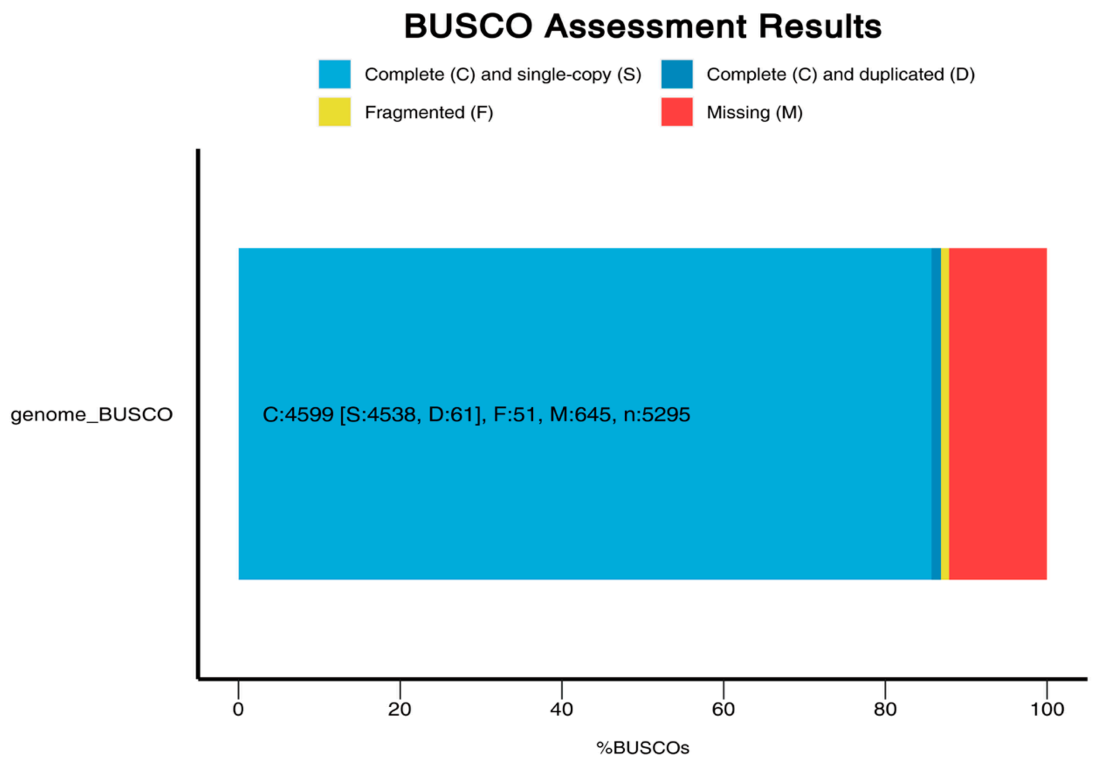

Figure S2. BUSCO completeness assessment o the final *S. angularis* genome assembly using the mollusca_odb10 lineage dataset.

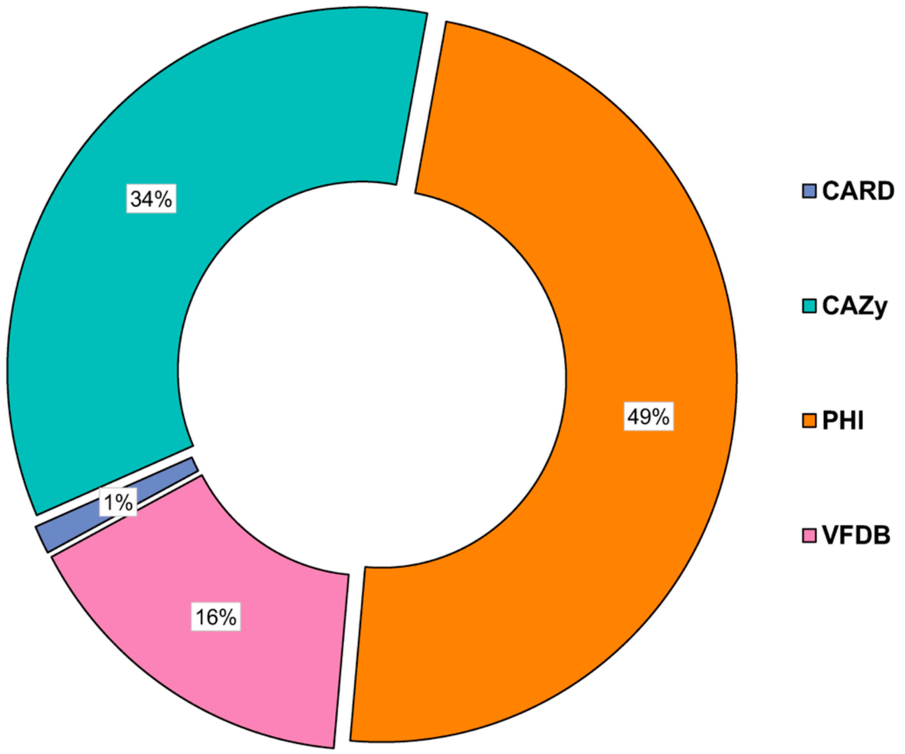

Figure S3. Distribution o predicted proteins annotated across our specialized unctional databases (PHI, CAZy, VFDB, and CARD).

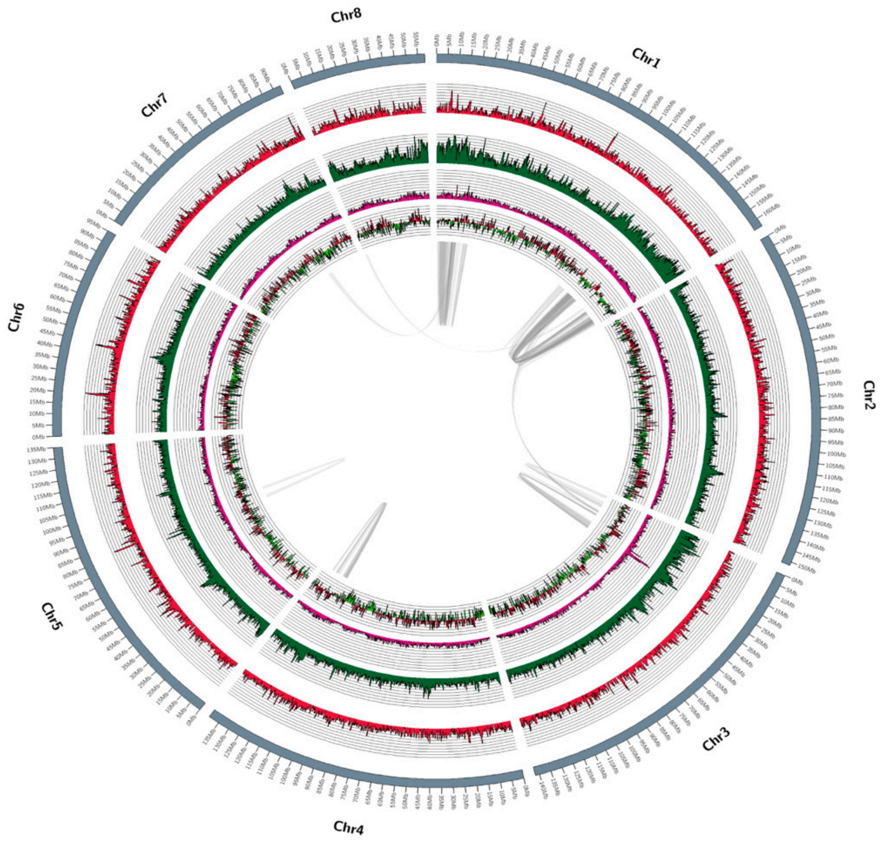

Figure S4. Circos plot illustrating the genome-wide landscape of genomic features across the eight chromosome-level scaffolds. Concentric tracks from outermost to innermost represent gene density, transposon density, repeat sequence density, GC content, and intra-genomic synteny blocks.
